# Susanne Kühl, Michael Kühl: Die Abschlussarbeit in den Life Sciences

**DOI:** 10.3205/zma001151

**Published:** 2018-02-15

**Authors:** Daniela Schmitz

**Affiliations:** 1 Universität Witten/Herdecke, Fakultät für Gesundheit, Lehrstuhl multiprofessionelle Versorgung chronisch kranker Menschen, Witten, Germany

## Recension

The book targets life sciences graduates. The authors intend to provide these graduates with a guideline for recognising and dealing with problems they may encounter when writing their thesis (page 9). Both authors work at the Institute of Biochemistry and Molecular Biology, Ulm University.

### Structure and contents

The book is subdivided into three main sections: a basic principles section (chapters 1 to 5), the main sections of a scientific thesis (chapters 6 to 11), and an exemplary thesis (chapter 12).

The **first chapter** provides general information on writing management, such as writing process phases, different writing types according to Scheuermann (2013), writing conditions on site, time and atmosphere, as well as writing problems, such as brainstorming, getting started and motivation, and approaches to solving these problems. This chapter also addresses the importance of data backup.

The **second chapter** deals with time management. It characterises different types of time, demonstrates the necessity of SMARTLY defined objectives, and illustrates the creation of schedules using the ALPEN method and various time management systems.

**Chapter three** focuses on the preparation and conception of the thesis and lists tips for literature research. Based on this, the essential characteristics and problems of the work phases, namely motivation, concept and structure, are described. Finally, chapter three discusses the importance of reference management and the selection of essential references.

The writing sequence up to the manuscript stage is explained by the **fourth chapter**. It illustrates the main process phases from raw text to chapter structure, from dividing the text into appropriate paragraphs to consolidating the central theme.

**Chapter five** presents the special features of scientific writing. It describes the need for aspects such as clarity of thought, precision, citation and explanation of intermediate steps.

The **sixth chapter** looks at the first main section of a thesis: the materials and methods section with its key elements and notes on wording.

The **seventh chapter** outlines the writing of the results section by taking a look at the logical structure, data editing, documentation and evaluation, as well as the visualisation of results by means of illustrations and tables.

Notes and guidelines for writing the introduction are summarised in **chapter eight**; inter alia, by dealing with the objective, relevance of the thesis, knowledge gaps, as well as with the research question and hypotheses, respectively.

**Chapter nine** focuses on key questions for writing the discussion section. Essential aspects of this chapter are the relevant topics of discussion, comparison with the current state of research and conclusions.

The **tenth chapter** establishes the key contents of the thesis summary and provides information on wording an appropriate and meaningful title.

**Chapter eleven** summarizes information on how to design the title page, table of contents, list of references, list of figures, appendix, etc. and provides tips as well as a checklist for refining the thesis.

**Chapter twelve** consists of an abridged version of an exemplary thesis having been compiled according to the guidelines of the previous chapters.

#### Discussion

The book illustrates the functions and required contents of the main sections of a thesis, and presents them along with text samples, tips on wording and key questions or design guidelines. The individual chapters are designed in a reader-friendly way, use checklists and information boxes, and provide practical tips, being complemented by helpful summaries at the end of each chapter. Moreover, the authors provide pragmatic tips for writing behaviour and self-motivation.

The publication focuses on experimental research theses and therefore omits other methodological designs in the methods chapters. The book consistently takes a thesis with an experimental approach as an example ("The function of XY during the eye development of Xenopus laevis") and uses this example to illustrate the requirements for writing scientific theses. The writing sequence is therefore also covered in a way specific to experimental practice. 

On the whole, the requirements and key contents of the methods, results and discussion sections are clearly defined in order to provide the reader with an orientation for his or her writing process.

#### Conclusion

This publication presents the essential contents and key requirements concerning the individual chapters of a thesis in life sciences. The materials and methods sections are specifically tailored to theses of an experimental nature. Various hints, writing and wording tips, sample texts and useful summaries at the end of each chapter are provided. The publication lives up to its claim of providing life sciences graduates with a guideline for writing experimental research theses.

## Competing interests

The author declares that she has no competing interests.

